# *In vitro* IL-6/IL-6R Trans-Signaling in Fibroblasts Releases Cytokines That May Be Linked to the Pathogenesis of IgG4-Related Disease

**DOI:** 10.3389/fimmu.2020.01272

**Published:** 2020-07-08

**Authors:** Ji Zongfei, Chen Rongyi, Cui Xiaomeng, Ma Lili, Ma Lingying, Kong Xiufang, Dai Xiaomin, Zhang Zhuojun, Chen Huiyong, Sun Ying, Jiang Lindi

**Affiliations:** ^1^Department of Rheumatology, Zhongshan Hospital, Fudan University, Shanghai, China; ^2^Evidence-Based Medicine Center, Fudan University, Shanghai, China

**Keywords:** IgG4-related disease, interleukin-6, fibroblasts, germinal center, T follicular helper cell, B cell, B cell activating factor

## Abstract

**Background:** The remarkable mechanisms of storiform fibrosis and the formation of high levels of IgG4 with a pathogenic germinal center (GC) in the inflammatory tissue of IgG4-RD remains unknown and may be responsible for the unsatisfactory therapeutic effect on IgG4-related diseases when using conventional therapy.

**Objectives:** To investigate the mechanisms of interleukin 6 (IL-6) inducing fibroblasts to produce cytokines for pathogenic GC formation in the development of IgG4-related disease (IgG4-RD).

**Methods:** The clinical data and laboratory examinations of 56 patients with IgG4-RD were collected. IL-6 and IL-6R expression in the serum and tissues of patients with IgG4-RD and healthy controls were detected by ELISA, immunohistochemistry, and immunofluorescence. Human aorta adventitial fibroblasts (AAFs) were cultured and stimulated with IL-6/IL-6 receptor (IL-6R). The effect of IL-6/IL-6R on AAFs was determined by Luminex assays.

**Results:** The serum IL-6 and IL-6R levels were elevated in active IgG4-RD patients and IL-6 was positively correlated with the disease activity (e.g., erythrocyte sedimentation rate [ESR], C-reactive protein [CRP], and IgG4-RD responder index). IL-6 and IL-6R expression in the tissue lesions of IgG4-related retroperitoneal fibrosis and IgG4-related sialadenitis patients were also significantly higher than that in the normal tissues. In addition, there is a relative abundance of myofibroblasts as well as IgG4^+^ plasma cells in the tissues of IgG4-related retroperitoneal fibrosis. α-SMA and B cell differentiation cytokines (i.e., B cell activating factor), and α-SMA and T follicular helper (Tfh) cell differentiation cytokines (e.g., IL-7, IL-12, and IL-23) were co-expressed in the local lesions. *In vitro*, IL-6/IL-6R significantly promoted the production of B cell activating factor, IL-7, IL-12, and IL-23 in AAFs in a dose-dependent manner. This effect was partially blocked by JAK1, JAK2, STAT3, and Akt inhibitors, respectively.

**Conclusions:** *In vitro* IL-6/IL-6R trans-signaling in fibroblasts releases Tfh and B cell differentiation factors partially via the JAK2/STAT3, JAK1/STAT3, and JAK2/Akt pathways, which may be linked to the pathogenesis of IgG4-RD. This indicated that IL-6 and fibroblasts may be responsible for GC formation and fibrosis in the development of IgG4-RD. Blocking IL-6 with JAK1/2 inhibitors or inhibiting fibroblast proliferation might be beneficial for IgG4-RD treatment.

## Introduction

IgG4-related disease (IgG4-RD) is a systematic, inflammatory, autoimmune disease (1). As a very new disease named only one decade ago, the number of IgG4-RD patients is increasing annually due to growing awareness (2). IgG4-RD typically involves multiple organs and mimics the clinical manifestations of a malignant tumor. Both a misdiagnosis or relapse of the disease will cause organ failure, including postrenal failure, liver cirrhosis, or an intestinal obstruction in affected patients (3). It has been well-established that one of the characteristic pathological features of this disease is remarkable storiform fibrosis (4), especially in patients with IgG4-related retroperitoneal fibrosis (IgG4-related RPF). Severe fibrosis of the retroperitoneum leads to hydronephrosis and postrenal failure, which contributes to the poor response to conventional treatment. Most IgG4-related RPF patients need Double J stent placement for a long time, with some even requiring life-long Double J stent treatment.

The patients with IgG4-RD usually receive glucocorticoids and immunosuppressors to relieve symptoms of the disease (5). However, it is well-known that, while glucocorticoids can alleviate inflammation rapidly, they cannot block the development of fibrogenesis in lesion tissues (6, 7). Therefore, the relapse rate of IgG4-RD is up to 24–54% during glucocorticoids tapering (4, 5), ultimately resulting in the high-dose, long-term use of glucocorticoids, which may lead to a high prevalence of adverse events (6). Recent studies found that B cell depletion treatment had a good curative effect in patients with IgG4-RD with a response rate of 93.5–97% (8, 9). However, the further efficacy and safety of rituximab still needs clinical verification. Thus, it is very important to explore the pathogenetic mechanisms of IgG4-RD in order to develop a more effective treatment strategy which will ultimately benefit patients.

The pathogenesis of IgG4-RD is rather complicated and still unclear. As the name of the disease implies, patients with IgG4-RD show elevated levels of the serum IgG4 accompanied with IgG4^+^ plasma cell infiltration in the inflammatory tissues (10–12). It is well-established that B cell activation and their differentiation into plasma cells play important roles in the development of IgG4-RD. Both the activation of Th2 and T follicular helper (Tfh) cells promote the activation and differentiation of B cells via Th2 cytokines (IL-4, IL-5, and IL-13) (13, 14) as well as Tfh cell cytokines (IL-21), respectively (15, 16). Local ectopic germinal center formation (GCF) is also one of the important pathological characteristics of IgG4-RD. It is well-known that local ectopic GCF is a key event in plasma cell differentiation, in which interlukin-6 (IL-6) and Tfh cells play a central role (12, 15, 16). With the stimulation of IL-6, Tfh cells could be activated and promote antibody secretion of B cells and its differentiation into plasma cells with the collaboration of BAFF, CD40L, IL-21, and IL-4 (17–22). In patients with IgG4-RD, a significantly increased number of Tfh cells was seen in the peripheral blood, tissue, tertiary lymphoid organs, and secondary lymphoid organs (18, 23–26). It was also found that Tfh cells were linked to IgG4 class switching (26) and could induce the differentiation of naïve B cells into plasmablasts and enhance the production of IgG4 in patients with active IgG4-RD (23). In addition, recent research indicated that an overexuberant repair process in cells, caused by B cells and Cytotoxic CD4 T cells, ultimately results in fibrosis and loss of target organ function in IgG4-RD (27). Therefore, further studies will be needed to identify the pathogenesis of IgG4-RD.

Recently, there have been an increasing number of studies showing that fibroblast is extremely important in the development of autoimmune diseases (28–30). In inflammatory tissues, fibroblasts produce various types of pro-inflammatory factors (e.g., IL-6 and IL-8) to promote the infiltration and activation of immune cells (31). Moreover, fibroblast proliferation could also be induced by cytokines (e.g., IL-6, IL-17, and TNF-α) (32), creating a self-feedback mechanism that aggravates tissue damage. However, whether and how this self-feedback mechanism is involved in IgG4-RD development remains unclear.

Interestingly, we have found remarkably higher levels of IL-6 and IL-6R expression in both the IgG4-related RPF and IgG4-related sialadenitis tissues. This indicated the activation of the IL-6/IL-6R signaling pathway in IgG4-RD. Thus, to explore how increased levels of IL-6/IL-6R promotes GCF-related cytokine production is extremely important. For signaling, IL-6 binds to the membrane-bound IL-6R (mIL-6R) or soluble IL-6R (sIL-6R) via glycoprotein 130 (gp130), referred to as classic or trans-signaling, respectively (33). IL-6R is mainly found on immune cells. In addition to the mIL-6R, sIL-6R is produced by proteolytic shedding and alternative splicing (34, 35).

In this study, we investigated the mechanisms by which IL-6 promotes GCF cytokine production in fibroblasts. Our results revealed that fibroblasts could produce IL-6, as well as Tfh cell and B cell differentiation factors, *in vitro*, partially via the JAK2/STAT3, JAK1/STAT3, and JAK2/Akt pathways. Thus, blocking the IL-6 pathway with JAK1/2 inhibitors or inhibiting fibroblast proliferation may be beneficial for IgG4-RD treatment.

## Materials and Methods

### Patients

We retrospectively recruited 56 IgG4-RD patients at Zhongshan hospital, Fudan University from Jan 1, 2016 to Dec 31, 2017. Among these patients, 42 were diagnosed as IgG4-RD by histopathological examination (36) and classified as definite IgG4-RD according to the 2012 Japanese classification criteria (37). The other 14 patients were diagnosed as possible IgG4-RD, clinically excluding carcinoma, infection, or other rheumatic disease, and responded to glucocorticoid treatment during a follow-up period of at least 1 year. Their general condition, clinical manifestation, level of erythrocyte sedimentation rate (ESR), C-reactive protein (CRP), serum IgG4, and serum cytokines (IL-6, IL-2R, TNF-α, and IL-8) were collected from the electronic medical records system. ESR was tested by ALIFAX TEST1 TH (Alifax) apparatus with a sensitivity of 2 mm/h. CRP was tested by CRP U-hs kit (Diasys) with a sensitivity of 0.03 mg/L. IgG4 was tested by OPAU03 (Siemens) with a sensitivity of 0.052 g/L. IL-6, IL-2R, TNF-α, and IL-8 were tested by LK6P1, LK8P1, IMMULITE 2000 IL2R, and LKNF1 (Siemens, chemiluminescence kit). Their sensitivities were 2 pg/ml, 5 U/ml, 1.7 pg/ml, and 2 pg/ml, respectively. All the above examinations were performed with standard procedures according to the instructions of the manufacturers by skilled technicians in our hospital. In addition, the level of serum IL-6 was also collected in 18 healthy people who received a medical checkup at our hospital from 2016 to 2017. The level of serum IL-6R was detected in the 56 patients with IgG4-RD and 20 healthy controls by ELISA (Abcam, ab46029).

The disease activity of IgG4-RD was measured via an IgG4-RD responder index (RI), according to the serum IgG4 concentration and affected organs (38). Patients were further divided into active and inactive groups as follows: (1) Major criteria—new onset or aggravating swelling or masses in single or multiple organs, confirmed by imaging examination; and (2) Minor criteria—(i) new symptom/signs related to IgG4-RD or a previous symptom/sign worsened during the last month, (ii) elevated levels of IgG4 or higher levels than that at the last follow-up, and (iii) involved organ failure or deterioration. If the patients satisfied one major criterion or more than one minor criterion in the list of criteria, they were defined as being in the active phase.

The retroperitoneum tissues were obtained from six patients with IgG4-related RPF and the salivary gland tissues from six patients with IgG4-related sialadenitis at Zhongshan hospital, Fudan University. The peri-tumoral retroperitoneum tissues were obtained from six patients with a retroperitoneum tumor and the control salivary glands tissues were collected from six patients with Sjogren's syndrome. The retroperitoneum tissues and salivary gland tissues were subjected to immunohistochemistry (IHC). The above-mentioned retroperitoneum tissues were subjected to double- and triple-labeled immunofluorescence staining.

The study protocol was approved by the Ethics Committees of Zhongshan Hospital [B2013-115(3)] and conforms to the ethical guidelines of the 1975 Declaration of Helsinki. All of the patients were required to provide informed consent.

### Immunohistochemistry

IHC was performed as previously described (39). PBS was used in the place of the primary antibody as the negative control. Briefly, after the sections were deparaffinized and rehydrated, endogenous peroxidase activity was blocked using 3% H_2_O_2_. Antigen retrieval was performed using an EDTA buffer solution (pH 8.0). Each slide was blocked with 1% BSA and incubated for 30 min. Subsequently, the slides were incubated with a diluted primary antibody for 1 h at 37°C and transferred from the incubator to 4°C overnight. The following day, the slides were warmed to room temperature and the secondary antibody was added to each slide for 1 h at 37°C. The sections were developed with DAB reagent, counterstained with hematoxylin, and mounted with neutral gum.

Anti-IL-6 and anti-IL-6R antibodies were purchased from Abcam (Cambridge, Mass). All slides were scanned with a 3DHISTECH scanner microscope and images were selected with a Pannoramic Viewer 1.15.3. The integrated optical density (IOD) and area of the selected images were determined using the Image Pro plus software v. 6.0 (Media Cybernetics, Silverspring, USA). Protein expression was presented as the ratio of IOD to the relevant area.

### Double- and Triple-Labeled Immunofluorescence

The procedures in this experiment were carried out as previously described (40). Briefly, the sections were deparaffinized and rehydrated, then antigen retrieval was performed using an EDTA antigen retrieval solution for 20 min at 95C−100°C. Each slide was blocked with 3% bovine serum albumin (BSA) for 30 min at room temperature. Subsequently, two or three types of diluted antibodies from different species were incubated with the slides for 1 h at 37°C and then 4°C overnight. The next day, the slides were incubated with species-specific fluorochrome conjugated secondary antibodies for 1 h at 37°C. The slides were counterstained with DAPI reagent and mounted with a medium to prevent quenching. The primary antibodies were all provided by Abcam. In double-labeled immunofluorescence (IF), we used the following antibodies:

1. Rabbit monoclonal anti-α-SMA (1:1,000) and rat monoclonal anti-BAFF (1:100) antibodies were used for α-SMA and BAFF dual IF.
2. Mouse monoclonal anti-α-SMA (1:1,000) and rabbit polyclonal anti-IL-7 (1:100) antibodies were used for α-SMA and IL-7 dual IF.
3. Rabbit monoclonal anti-α-SMA (1:1,000) and rat monoclonal IL-12 (1:100) antibodies were used for α-SMA and IL-12 dual IF.
4. Mouse monoclonal anti-α-SMA (1:1,000) and rabbit polyclonal anti-IL-23 (1:100) antibodies were used for α-SMA and IL-23 dual IF.
5. The secondary antibodies were comprised of Alexa Fluor 488-conjugated goat anti-rabbit and Cy3-conjugated goat anti-mouse antibodies, Cy3-conjugated goat anti-rabbit antibodies (Beyotime Biotechnology, China, dilution multiple 1:500) and FITC-AffiniPure Goat Anti-Rat antibody (dilution multiple 1:100, Jaskon, American).

In the triple-labeled IF, primary antibodies, including goat polyclonal anti-α-SMA, rabbit monoclonal anti-IL-6, and mouse monoclonal anti-IgG4 antibodies, were used. The secondary antibodies were comprised of Alexa Fluor 488-conjugated donkey anti-goat, Alexa Fluor 594-conjugated donkey anti-rabbit, and Alexa Fluor 647-conjugated donkey anti-mouse antibodies (Jackson, American) and they were all diluted to 200 times before use. The samples were scanned using a laser scanning confocal microscope (Olympus, FV-1000, Japan). Protein expression was presented as the ratio of IOD to the relevant area.

### Cell Culture

To date, it has been difficult to obtain a biopsy or perform minimally invasive surgery to collect aortic adventitial fibroblasts (AAFs) from patients with IgG4-related RPF. Therefore, we used AAF derived from healthy individuals for this *in vitro* study.

In the current study, human AAFs (catalog no. 6120, AAF) were purchased from Sciencell Research Laboratories (Corte Del Cedro, Carlsbad CA, USA). AAFs were cultured in Dulbecco's modification of Eagle's medium (DMEM) supplemented with 10% fetal bovine serum. IL-6/IL-6R were purchased from R&D, Minneapolis, MN, USA. The AAF culture supernatant was collected from these experiments and frozen at −80°C for a Magnetic Luminex Assay to measure the level of IL-7, IL-12, IL-23, and BAFF (R&D Systems, Minneapolis, MN, USA).

In this study, different concentrations of IL-6/IL-6R (0, 10, 20, 50, and 100 ng/ml) were used to stimulate AAFs and the levels of BAFF, IL-7, IL-12 p70, and IL-23 of supernatant were detected at different time points (24 and 48 h) to observe the effects of IL-6/IL-6R on AAFs. In addition, the concentration of the above cytokines following an intervention with a signal inhibitor were explored at different time points (24 and 48 h). The JAK1 inhibitor (Itacitinib, 10 ng/ml), JAK2 inhibitor (AG490), STAT3 inhibitor (S31-201), and Akt inhibitor (LY294002) were used to pretreat AAFs to explore the mechanism by which IL-6/IL-6R induces the production of BAFF, IL-7, IL-12 p70, and IL-23 in AAFs.

### Statistical Analysis

The continuous variables were summarized as: the means ± standard deviation for the normal distributions in the clinical information and histopathological data; and the means ± standard error for the normal distributions in the *in vitro* experiment. Categorical variables were described as numbers and percentages. A one-way analysis of variance (ANOVA), Bonferroni *post hoc* tests, and a Mann-Whitney U test were used, as applicable, for comparisons between groups. A two-tailed *p* < 0.05 was considered statistically significant. Statistical analysis was performed using SPSS for Windows, Version 20.0 (IBM Corp., Armonk, NY, USA).

## Results

### IL-6 and IL-6R Were Elevated in Both the Serum and Tissue of Patients With IgG4-RD

**Figure 1:**
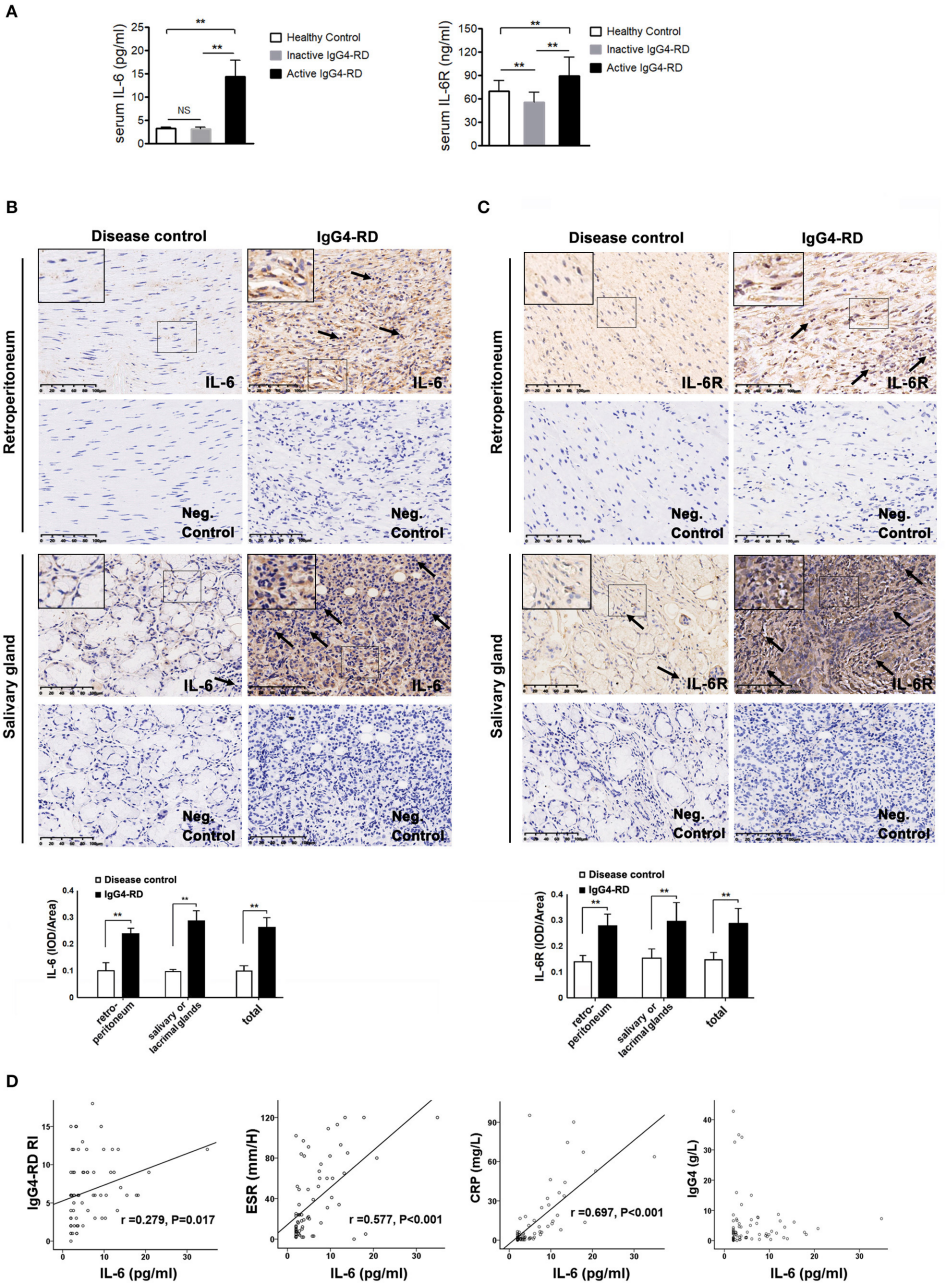
The level of IL-6 and IL-6R in the serum and tissues of IgG4-RD and the correlation between serum IL-6 and disease activity. **(A)** The level of serum IL-6 (*N* = 74) and IL-6R (*N* = 76) was significantly higher in the active IgG4-RD patients than those in the inactive patients, as well as that of the healthy controls (*p* < 0.01). Compared with the healthy controls, the inactive patients exhibited even lower levels of IL-6R (*p* < 0.01). **(B,C)** The salivary gland tissue was comprised of glandular tube and acinus. Large amounts of lymphocytes and plasmocytes infiltrated the IgG4-related sialadenitis tissue (←), which was in contrast with the small number of lymphocytes observed in the disease control tissue (←). The control retroperitoneum tissue was comprised of fibrous tissue. However, the IgG4-related RPF tissue was filled with lymphocytes and plasmocytes (←), while few lymphocytes were observed in the control tissue. The negative (neg.) controls did not show interference. The immunochemistry results exhibited higher IL-6 and IL-6R expression in both the IgG4-related RPF and IgG4-related sialadenitis tissues compared to the control tissues, which was confirmed by a semi-quantitative analysis (*n* = 6) for each group. **(D)** The level of serum IL-6 showed a positive correlation to ESR, CRP, and IgG4-RD RI scores in the patients with IgG4-RD (n = 56), but was not correlated with serum IgG4. **p < 0.01.

A total of 56 patients with IgG4-RD were enrolled in the present study (male to female ratio: 47:9). The mean age of disease onset was 57.4 ± 14.4 years old. There were 40 patients in the active phase and 16 patients in the inactive phase. The associated clinical information is listed in Table 1. The level of serum IL-6 and IL-6R was significantly higher in the active patients compared to that in the inactive patients and those in the healthy controls (Figure 1A); however, the other cytokine levels (e.g., TNF-α, IL-2R, and IL-8) were not significantly different between these two groups (Table 1). Among the 56 patients, 28 patients (50%) displayed retroperitoneum involvement. Among the 28 patients with IgG4-related RPF, the levels of serum IL-2R and IL-6 were elevated in the active group (Table 1 and Figure 1A). The level of serum IL-6 was also positively correlated with ESR, CRP, and the IgG4-RD RI score in all 56 patients with IgG4-RD (Figure 1D). However, there was no correlation between the level of serum IL-6 and serum IgG4. The immunochemical analysis of the retroperitoneum tissue revealed higher IL-6 and IL-6R expression in both the tissue of IgG4-related RPF and IgG4-related sialadenitis than in the control tissue (Figures 1B,C).

**Table 1:** Clinical information and examinations in IgG4-RD patients.

	**All IgG4-RD (*n* = 56)**	**Active IgG4-RD (*n* = 40)**	**Inactive IgG4-RD (*n* = 16)**	**All IgG4-related RPF (*n* = 28)**	**Active IgG4-related RPF (*n* = 21)**	**Inactive IgG4-related RPF (*n* = 7)**
Sex (M/F)	47/9	33/7	14/2	25/3	18/3	7/0
onset Age	57.35 ± 14.43	56.11 ± 14.41	60.31 ± 14.53	57.44 ± 14.97	55.95 ± 15.1	61.71 ± 14.82
Number of involved organs	2.24 ± 1.23	2.21 ± 1.22	2.31 ± 1.30	2.18 ± 1.25	2.00 ± 1.23	2.71 ± 1.25
IgG4-RD RI	7.13 ± 4.34	8.97 ± 3.81^**^	2.63 ± 1.46	7.04 ± 4.34	8.48 ± 4.03^**^	2.71 ± 1.25
Serum IgG4 (g/L)	3.89 ± 6.11	4.62 ± 6.74^**^	2.12 ± 3.83	3.01 ± 2.35	3.74 ± 2.80^**^	0.82 ± 0.38
ESR (mm/h)	43.75 ± 38.57	57.54 ± 37.94^**^	11.88 ± 12.23	48.11 ± 39.48	60.48 ± 38.06^**^	11.00 ± 6.56
CRP (mg/L)	17.62 ± 25.12	23.91 ± 27.62^**^	2.69 ± 3.19	15.03 ± 24.96	25.20 ± 29.97^*^	3.56 ± 4.08
Serum TNF-α (pg/ml)	16.98 ± 36.69	18.63 ± 42.70	12.99 ± 15.01	12.76 ± 7.80	23.98 ± 60.09	15.32 ± 22.23
Serum IL-2R (pg/ml)	751.94 ± 603.06	851.39 ± 679.78	528.19 ± 284.86	657.84 ± 368.34	740.89 ± 392.65^*^	444.29 ± 178.86
Serum IL-6 (pg/ml)	11.00 ± 18.94	14.38 ± 21.43^**^	3.02 ± 1.51	9.90 ± 17.59	12.10 ± 19.90^*^	3.33 ± 2.21
Serum IL-6R (ng/ml)	73.89 ± 25.59	89.24 ± 23.92^**^	55.83 ± 12.38	64.54 ± 13.22	73.80 ± 7.23^*^	57.93 ± 12.78
Serum IL-8 (pg/ml)	20.73 ± 26.70	17.78 ± 21.72	27.36 ± 35.43	19.06 ± 30.01	25.96 ± 43.38	16.36 ± 24.03

* p < 0.05 vs. inactive group;

** *p < 0.01 vs. inactive group*.

*IgG4-RD, IgG4-related disease; IgG4-related RPF, IgG4-related retroperitoneal fibrosis*.

### IL-6-Producing Fibroblasts and IgG4^+^ Plasma Cells in the Tissue Lesions of IgG4-Related RPF

**Figure 2:**
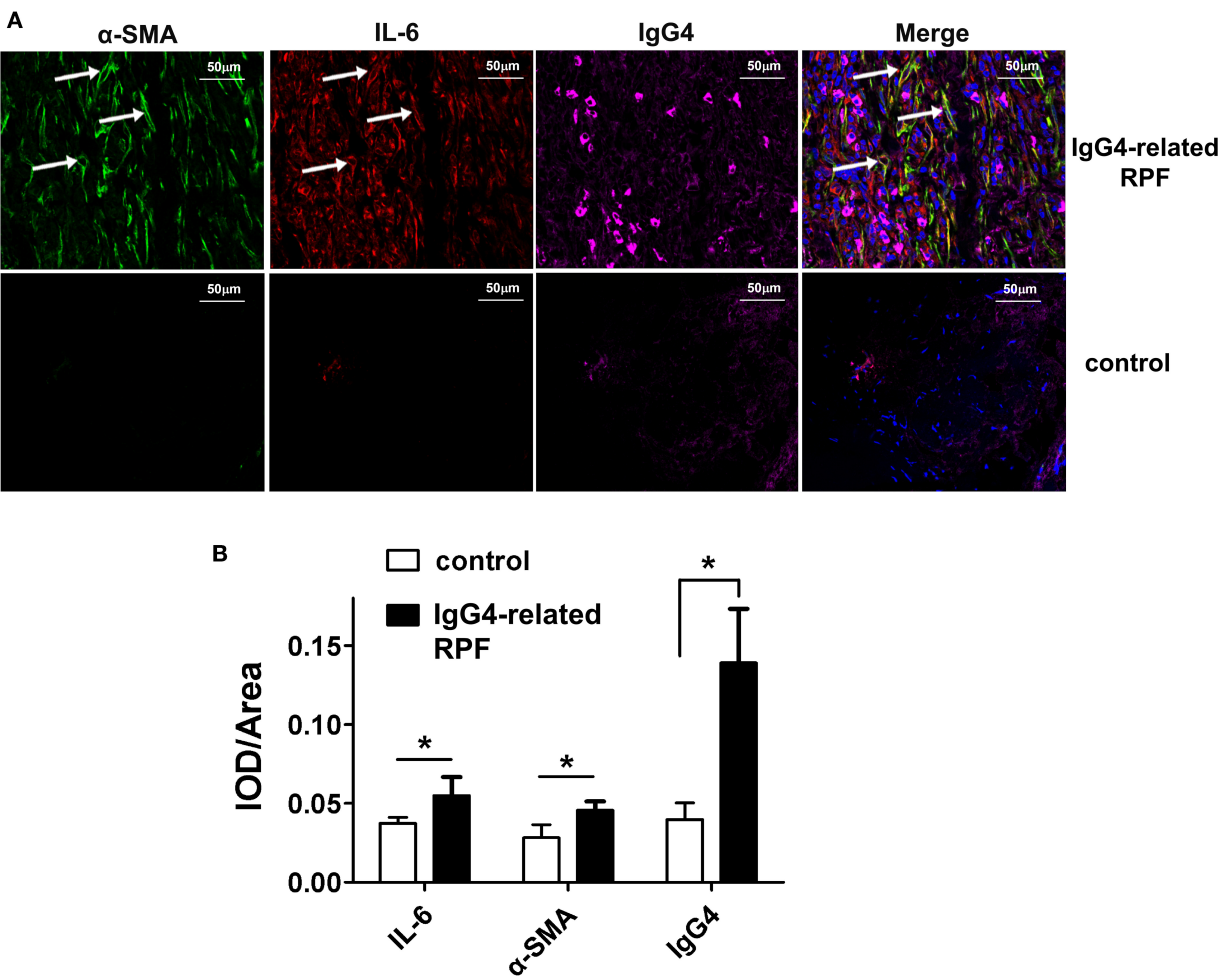
Representative triple-labeled immunofluorescence images in the IgG4-related RPF tissues. **(A)** The expression of IL-6, α-SMA, and IgG4 in the lesion tissues and controls are shown. The white arrow indicates IL-6-producing myofibroblasts (*p < 0.05; *n* = 6). **(B)** The relative expression of the proteins was determined using the ratio of IOD (integrated optical density) to area.

The results showed the high expression of IL-6, relative abundance myofibroblasts, and IgG4^+^ plasma cells (Figure 2 and Figure S1) in the tissue lesions of IgG4-related RPF patients, which were confirmed by both the triple-stained immunofluorescence and immunohistochemistry assay. In addition, the results indicated an increase of IL-6-producing myofibroblasts (Figure 2, arrows). In contrast, no similar phenomenon was observed in the control peri-tumoral retroperitoneum tissue. Therefore, high IL-6 expression and IL-6-producing fibroblasts may contribute to the infiltration of IgG4^+^ plasma cells.

### Exogenous IL-6/IL-6R Promotes Fibroblast Proliferation and the Production of Tfh Cell and B Cell Differentiation Factors

**Figure 3:**
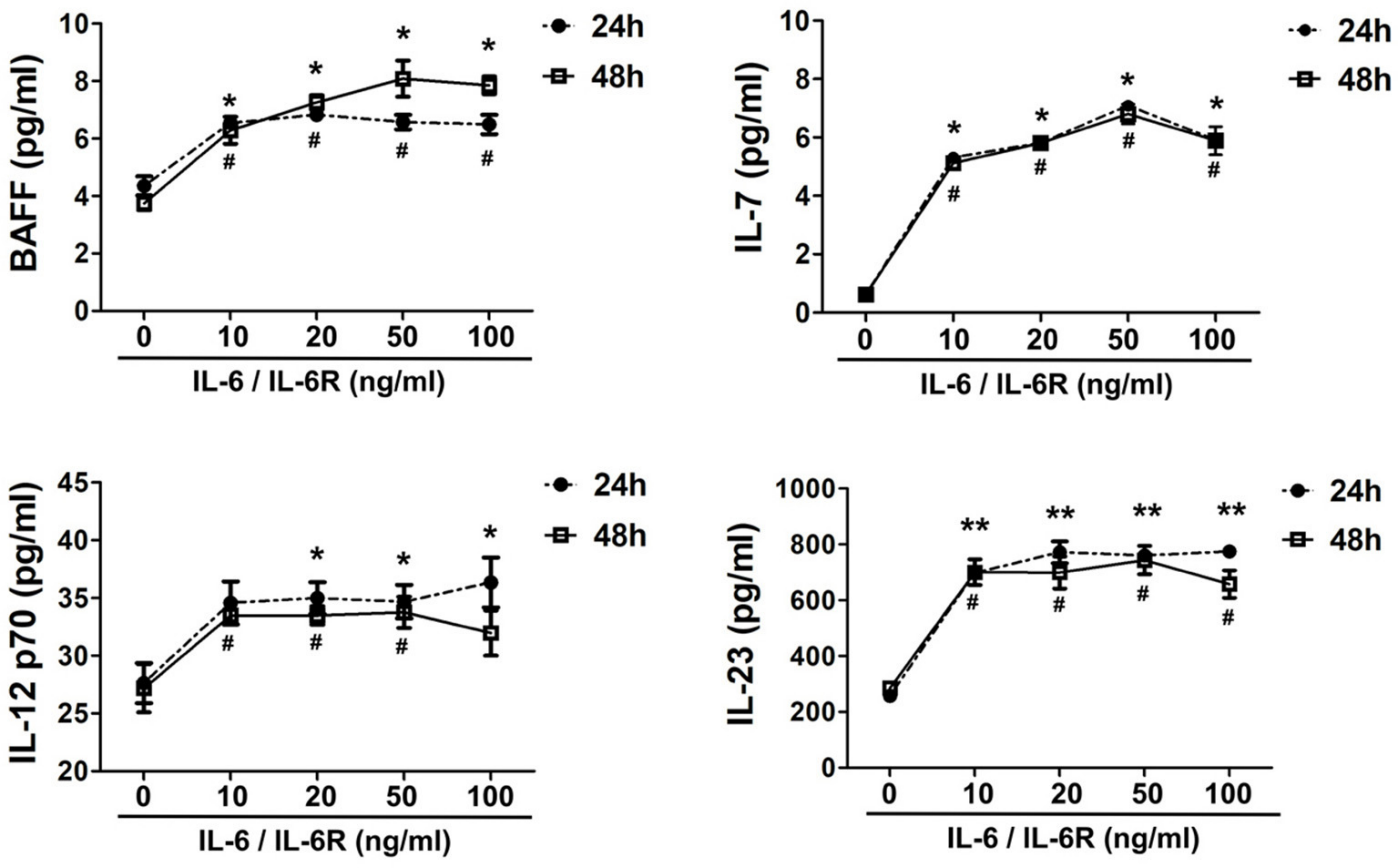
Exogenous IL-6/IL-6R promotes the production of Tfh cell and B cell differentiation factors. The secretion of cytokines (IL-7, BAFF, IL-12 p70, and IL-23) in the supernatant after stimulation of IL-6/IL-6R at 24 h and 48 h (*n* = 6). *p < 0.05 vs. control group (24 h); **p < 0.01 vs. control group (24 h); ^**#**^*p* < 0.05 vs. control group (48 h).

IL-6R is mainly produced by multiple inflammatory cells (e.g., macrophages, neutrophils, and lymphocytes). Thus, exogenous IL-6R should be added in the in *vitro* experiments when IL-6 is used to stimulate AAFs due to the lack of IL-6R expression in fibroblasts (41). Following IL-6/IL-6R activation, the secretion of IL-7, BAFF, IL-12 p70, and IL-23 increased significantly in the cellular supernatant (Figure 3), at both 24 and 48 h by a Luminex assay (*p* < 0.05), especially at an IL-6/IL-6R dose of 50 ng/mL, indicating that 50 ng was the optimal dose for the *in vitro* experiments. It has been well-established that IL-7 can induce T cell survival and maturity (42–44). Furthermore, IL-7 (45–47), IL-12 (48–51), and IL-23 (12, 48, 52) are responsible for Tfh cell differentiation. Thus, with the combined effects of Tfh cells and BAFF on B cells, B cells may finally differentiate into plasma cells and produce high level of IgG4.

### Fibroblasts Produce Tfh Cell and B Cell Differentiation Factors in IgG4-Related RPF Patients

**Figure 4:**
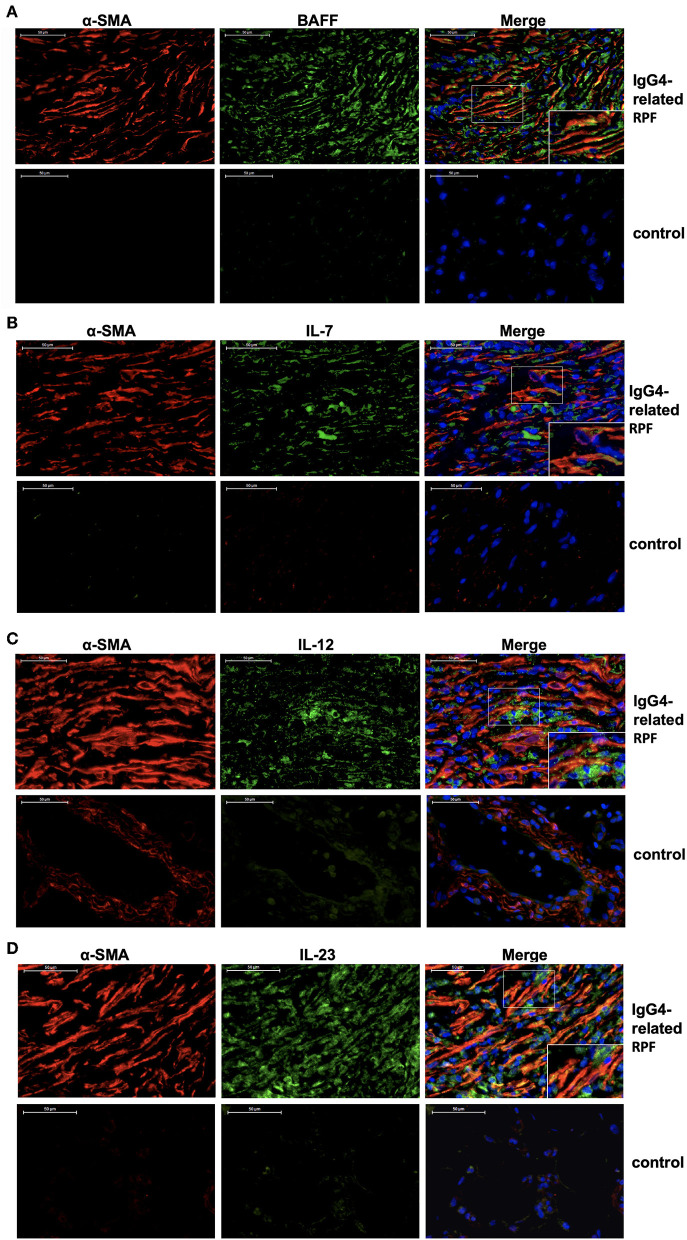
Fibroblasts produce Tfh cell and B cell differentiation factors in IgG4-related RPF patients. Representative double-labeled immunofluorescence images of a-SMA and BAFF **(A)**; α-SMA and IL-7 **(B)**; α-SMA and IL-12 p70 **(C)**; α-SMA and IL-23 **(D)** in the retroperitoneum tissues of IgG4-related RPF and the control retroperitoneum tissues is shown (*n* = 6).

Double-labeled fluorescence staining revealed the co-expression of α-SMA and BAFF, α-SMA and IL-7, α-SMA and IL-12 p70, as well as α-SMA and IL-23 in the retroperitoneum of IgG4-related RPF patients compared to the control tissue (Figure 4). These results suggest that the activated fibroblasts in the retroperitoneum tissue may express BAFF, IL-7, IL-12 p70, and IL-23 in patients with IgG4-related RPF.

### IL-6/IL-6R Promotes Fibroblast Production of Tfh Cell and B Cell Differentiation Factors in AAFs Partially Through the JAK1/STAT3, JAK2/STAT3, and JAK2/Akt Pathways

**Figure 5:**
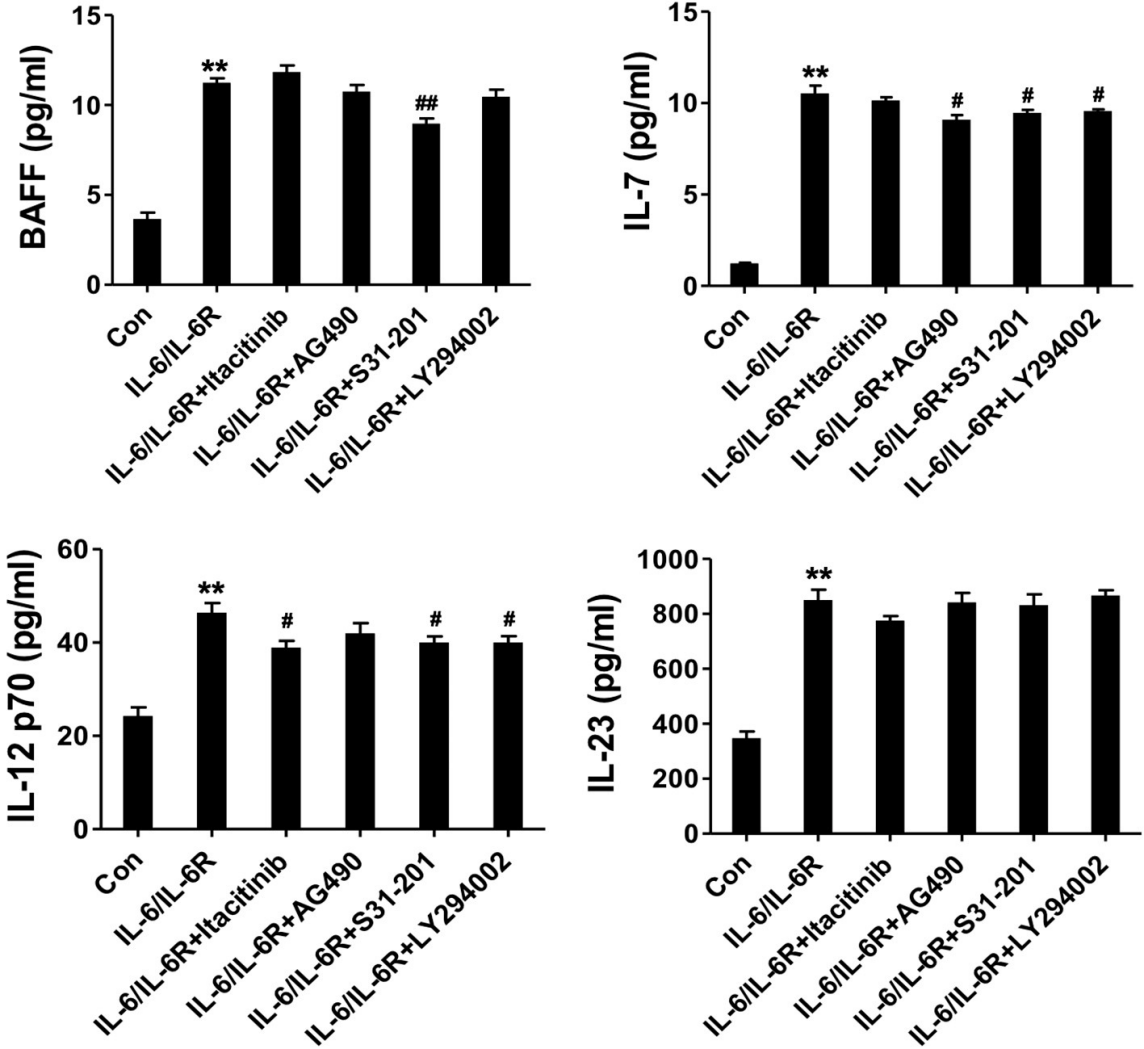
The secretion of cytokines (IL-7, BAFF, IL-12 p70, and IL-23) in the supernatant following stimulation with IL-6/IL-6R (50 ng/ml) with or without different inhibitors; **p < 0.01 vs. the control group; ^#^*p* < 0.05 vs. IL-6/IL-6R group; ^*##*^*p* < 0.01 vs. IL-6/IL-6R group; JAK1 inhibitor = Itacitinib; JAK2 inhibitor = AG490; STAT3 inhibitor = S31-201; Akt inhibitor = LY294002.

To address the IL-6-dependent production of the cytokine panel, we pretreated the AAFs with the respective signaling pathway inhibitors and examined the altered expression profiles. The results showed that BAFF, IL-7, and IL-12 p70 were all significantly reduced in the presence of the STAT3 inhibitor. IL-7 expression was significantly inhibited in the presence of the JAK2 and Akt inhibitors. IL-12 p70 was also decreased in the presence of the JAK1 and Akt inhibitors (Figure 5). In contrast, IL-23 expression was not affected by these inhibitors. Our data indicate that IL-6/IL-6R induced the production of Tfh cell and B cell differentiation factors in AAFs, partially via the JAK1/STAT3, JAK2/STAT3, and JAK2/Akt pathways.

## Discussion

It is believed that IL-6 directly promotes the development of fibrosis in damaged tissues (41, 53); however, whether IL-6 contributes to the pathogenesis of IgG4-RD remains unknown. In this study, we found for the first time that the level of IL-6 and IL-6R were increased in both the serum and tissue of patients with IgG4-RD. Further analysis revealed that the increased level of serum IL-6 was positively correlated with ESR, CRP, and the disease activity assessment score (e.g., IgG4-RD RI), but not with serum IgG4 in IgG4-RD patients. Indeed, IgG4-RD RI, rather than serum IgG4, is closely related to disease activity (23, 38), indicating that the increased level of IL-6 was directly correlated with disease activity and was also involved in the development of IgG4-RD.

As a critical pro-inflammatory factor, IL-6 is produced by both immune cells, as well as various types of tissue cells (e.g., fibroblasts, epithelial cells, and keratinocytes) under the stimulation of IL-1, TNF-α, PDGF, virus, double-stranded RNA, and c-AMP, and subsequently promotes the development of inflammatory and autoimmune diseases (54–58). However, whether tissue cells in the affected organs of IgG4-RD can produce IL-6 remains unclear. In this study, immunofluorescence triple-staining and an immunohistochemistry assay detected multiple IL-6-producing myofibroblasts in the affected tissues of IgG4-RD patients. This finding indicates that IL-6, which is partially synthesized by myofibroblasts, might be an initiator for the development of IgG4-RD under an inflammatory microenvironment.

As the main cellular components of connective tissues, fibroblasts act as supporting cells, but also play a critical role in the pathogenesis of inflammatory and autoimmune diseases (28–30). For example, in rheumatoid arthritis, synovial fibroblasts stimulated with IL-17 and Cyr61 could over-proliferate (59) and produce IL-6 and IL-8. This ultimately resulted in synovial hyperplasia, further Th17 differentiation, and macrophage infiltration (31, 60). Similarly, skin fibroblasts and keratinocytes have been found to secrete proinflammatory cytokines and promote inflammation in some disorders (e.g., psoriasis) (61). It has also been confirmed that IL-6 could be over-expressed in AAFs induced by IL-6 via the JAK2/STAT3 and JAK2/AKT pathways (41). Thus, fibroblasts are considered to be an important therapeutic target cell in a series of inflammatory and autoimmune diseases, including rheumatoid arthritis and Graves' ophthalmopathy (62, 63). Our results indicate the use of fibroblasts might be a novel therapeutic target in IgG4-RD, which is consistent with the phenomenon that IL-6-producing fibroblasts exist in the submandibular glands of patients with IgG4-RD (64).

It has been well-established that substantial IgG4^+^ plasma cell infiltration is a fundamental feature in the pathologic changes of IgG4-RD (7, 36, 37). The local ectopic GCF consists of Tfh cells, B cells, and plasma cells, which are commonly observed in the affected tissues of IgG4-RD (10, 36, 65). Tfh cells were found to promote the maturation, differentiation, and IgG4 production of surrounding B cells via IL-21 and IL-4 (17). B cells subsequently differentiated into plasma cells in the presence of BAFF (20, 21). As expected, the increased number of Tfh cells was positively correlated with the disease activity of IgG4-RD (18, 19). However, whether and how fibroblast produce Tfh and plasma cell differentiation factors have not been previously reported in the pathogenesis of IgG4-RD.

To further explore these gaps in the literature, we added exogenous IL-6/IL-6R to stimulate human AAFs, a type of fibroblast located in the wall of the aorta, which closely adjoins the lesions of IgG4-related RPF. These results show that the AAFs expressed high concentrations of cytokines for promoting Tfh cell differentiation (e.g., IL-7, IL-12 p70, and IL-23) and plasma cell differentiation (BAFF) in a dose-dependent manner. Our results strongly indicate that in IgG4-RD development, once fibroblasts are activated by IL-6, they can promote IgG4-RD development by simultaneously producing both IL-6 and Tfh/B cell differentiation factors in a malicious self-feedback manner. This finding was further supported by the *in vivo* results with immunofluorescence in the retroperitoneum tissues of IgG4-related RPF patients in the current study.

Our study indicates that IL-6/IL-6R trans-signaling in fibroblasts releases cytokines that may be linked to the pathogenesis of IgG4-RD. IL-6 stimulation promotes the proliferation and production of collagen and fibronectin by fibroblasts (41), and consequently induces lesion tissue fibrosis and even loss of function. In turn, over-proliferative fibroblasts may produce various kinds of cytokines which further promote Tfh/B cell differentiation and GCF, which may further result in IgG4-secreting plasma cell infiltration, aggravated inflammation, tissue damage, and fibrosis in a malicious self-feedback manner. However, this speculation is mainly based on our *in vitro* studies. Numerous internal factors could have influences on the development of IgG4-RD *in vivo*. Further *in vivo* research might be needed to confirm the role of IL-6 and fibroblasts in the pathogenesis of this disease.

It has been well-established that glucocorticoids and immunosuppressants are the primary reagents used in the treatment of IgG4-RD, which have been shown to effectively inhibit the proliferation of immune cells, thereby rapidly reducing inflammation. However, the current treatment could not block fibrosis and tissue damage, because tissue cells (e.g., fibroblasts and myofibroblasts) are not sensitive to these reagents. Thus, it is important to develop a novel means of inhibiting both fibroblast proliferation and IL-6 production.

In the current study, we investigated the signaling pathways involved in the production of Tfh/B cell differentiation-related cytokines in fibroblasts induced by IL-6. The results showed that IL-7, IL-12 p70, IL-23, and BAFF expressed in AAFs could be partially blocked by JAK1, JAK2, STAT3, and AKT inhibitors. JAK inhibitors have demonstrated a remarkable therapeutic effect on rheumatic arthritis (66) and many other immune-mediated diseases, including systemic lupus erythematosus (SLE) (67), and dermatomyositis (68). The use of JAK inhibitors in IgG4-RD treatment should be studied in the future.

To date, anti-IL-6 therapy has been widely used to treat autoimmune diseases, including rheumatic arthritis (69), giant cell arteritis (70), and adult-onset Still's disease (AOSD) (71). Our results suggest that blocking IL-6 with JAK1/2 inhibitors or inhibiting fibroblast proliferation might represent a beneficial IgG4-RD treatment.

## Conclusions

With the stimulation of IL-6/IL-6R, fibroblasts can produce IL-6 and further produce Tfh and B cell differentiation factors *in vitro*, partially via the JAK2/STAT3, JAK1/STAT3, and JAK2/Akt pathways. These findings indicate that IL-6/IL-6R trans-signaling in fibroblasts releases cytokines that may be linked to the pathogenesis of IgG4-RD. Therefore, blocking IL-6 with JAK1/2 inhibitors or inhibiting fibroblast proliferation might be a beneficial treatment for IgG4-RD.

## Data Availability Statement

All datasets generated for this study are included in the article/Supplementary Material.

## Ethics Statement

The studies involving human participants were reviewed and approved by the Ethics Committees of Zhongshan Hospital. The patients/participants provided their written informed consent to participate in this study.

## Author Contributions

JZ and CR conceived of, designed, and performed the experiments. MLin, DX, ZZ, and CH collected the clinical data and samples. CX and MLil analyzed the data. JZ, JL, and SY contributed to writing the manuscript. All of the authors reviewed and approved the final manuscript.

## Conflict of Interest

The authors declare that the research was conducted in the absence of any commercial or financial relationships that could be construed as a potential conflict of interest.

## Supplementary Material

Figure S1 Serial sections of IgG4-related RPF tissue showed lymphocyte and plasmacyte infiltration along with IL-6, IgG4, and α-SMA expression. **(A)** HE staining exhibited diffuse lymphocyte and plasmacyte (←) infiltration in the IgG4-related RPF tissue, as well as collagenous fiber proliferation (▴). **(B)** IgG4^+^ plasma cells were observed in the IgG4-related RPF tissue by immunochemistry examination (upper right, ←). **(C)** The immunochemistry results exhibited extensive positive IL-6 expression in the IgG4-related RPF tissue, which could be expressed by lymphoplasmacytes (←) and myofibroblasts (□). **(D)** The IL-6-expressing myofibroblasts were confirmed by an α-SMA (□) immunofluorescence test in the adjacent section of the IgG4-related RPF tissue.
