# Supplementary figures and images for: In vitro IL-6/IL-6R Trans-Signaling in Fibroblasts Releases Cytokines That May Be Linked to the Pathogenesis of IgG4-Related Disease
Source: Front Immunol. 2020 Jul 8;11:1272. doi: 10.3389/fimmu.2020.01272 (PMC7360847; doi:10.3389/fimmu.2020.01272)

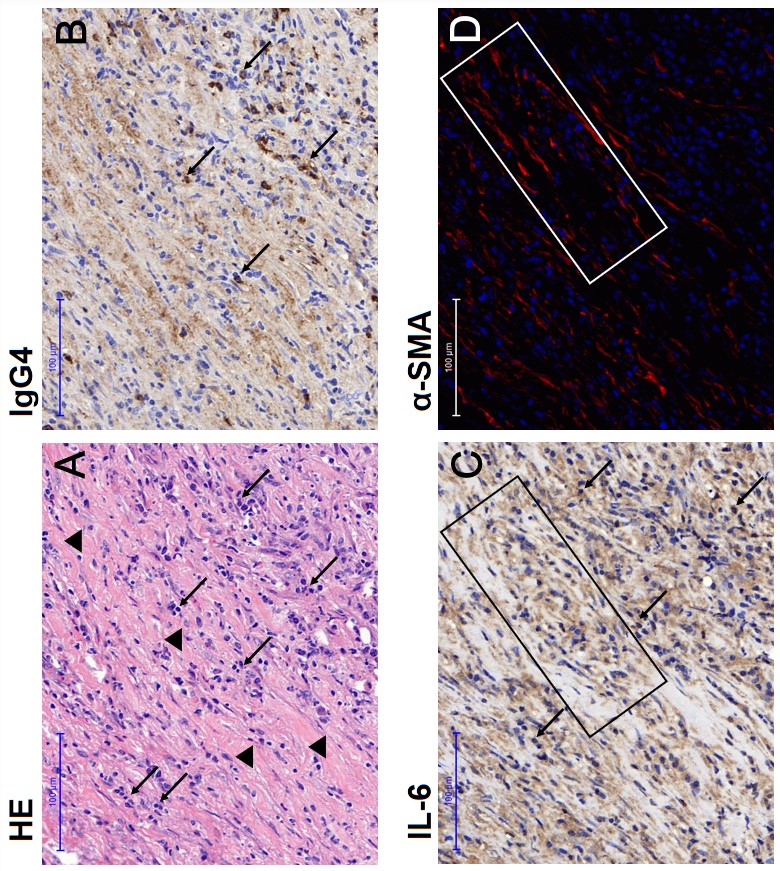

Supplement: Figure S1 — Serial sections of IgG4-related RPF tissue showed lymphocyte and plasmacyte infiltration along with IL-6, IgG4, and α-SMA expression. (A) HE staining exhibited diffuse lymphocyte and plasmacyte (←) infiltration in the IgG4-related RPF tissue, as well as collagenous fiber proliferation (▴). (B) IgG4+ plasma cells were observed in the IgG4-related RPF tissue by immunochemistry examination (upper right, ←). (C) The immunochemistry results exhibited extensive positive IL-6 expression in the IgG4-related RPF tissue, which could be expressed by lymphoplasmacytes (←) and myofibroblasts (□). (D) The IL-6-expressing myofibroblasts were confirmed by an α-SMA (□) immunofluorescence test in the adjacent section of the IgG4-related RPF tissue. [file Image_1.JPEG]
